# Theoretical study of the oxidation reactions of sulfurous acid/sulfite with ozone to produce sulfuric acid/sulfate with atmospheric implications†

**DOI:** 10.1039/c8ra00411k

**Published:** 2018-02-20

**Authors:** Fang Sheng, Liu Jingjing, Chen Yu, Tao Fu-Ming, Duan Xuemei, Liu Jing-yao

**Affiliations:** Institute of Theoretical Chemistry, Laboratory of Theoretical and Computational Chemistry, Jilin University Changchun 130023 China duanxm@jlu.edu.cn; Department of Chemistry, Key Laboratory of Organic Optoelectronics & Molecular Engineering of Ministry of Education, Tsinghua University Beijing 100084 China enouragement@mail.tsinghua.edu.cn; Department of Chemistry and Biochemistry, California State University Fullerton California 92834 USA

## Abstract

Herein, theoretical studies were performed on the atmospheric oxidation of sulfurous acid (H_2_SO_3_) and sulfite ions (HSO_3_^−^) by ozone (O_3_) to produce sulfuric acid and hydrosulfate ions. The most favorable path for the H_2_SO_3_ + O_3_ reaction has been found to be initiated from concerted H-abstraction and oxygen addition, with an overall energy barrier of 18.3 kcal mol^−1^. On the other hand, the most favorable path for the HSO_3_^−^ + O_3_ reaction is initiated from oxygen addition, with an overall energy barrier of only 0.3 kcal mol^−1^. Kinetic simulations were performed to estimate the significance of these reactions in the formation of atmospheric sulfate and destruction of the ozone layer. The results provide new insight into the missing source of atmospheric sulfate and particulate matter.

## Abstract 2

Herein, theoretical studies were performed on the atmospheric oxidation of sulfurous acid (H_2_SO_3_) and sulfite ions (HSO_3_^−^) by ozone (O_3_) to produce sulfuric acid and hydrosulfate ions.

## Graphical abstract

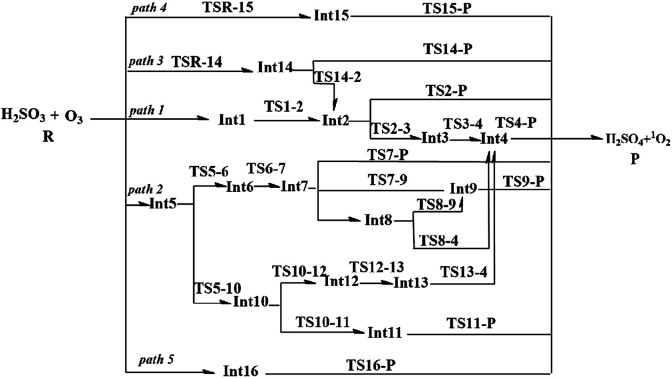

## 1. Introduction

Oxidation reactions are the most important reactions in the atmosphere, which connect anthropogenic and natural species.^1,2^ The oxidation of sulfur species is considered to be the main channel for the production of atmospheric sulfuric acid (H_2_SO_4_) or sulfate species.^3,4^ Gaseous sulfuric acid and sulfate have been identified as the major drivers to generate atmospheric aerosols.^3,5–7^ As is well known, aerosols have a significant impact on the global environment such as climate change, reduction in visibility, and public health effects.^8^ In addition, hexavalent sulfur species are confirmed as the major fraction of PM2.5 (aerosol particles with an aerodynamic diameter less than 2.5 μm), which are associated with certain sicknesses and other acute or chronic health effects.^7^ Air pollution caused by sulfur emissions will remain for a long time in China due to its coal-based energy structure.^9^ Haze days with high concentrations of PM2.5 appear frequently in the northern cities of China during the cold winter and spring seasons^10^ due to the increase in emissions from heating.^11,12^ Atmospheric aerosols are hazardous to both human health and the environment.^3,7,8^ Therefore, understanding the formation process of sulfuric acid or sulfate species is a critical step in developing the atmospheric sulfur cycle.

The oxidation of sulfur dioxide (SO_2_) is known as the main source of atmospheric sulfate.^4,13–15^ The oxidation reactions of SO_2_ have been extensively studied in a variety of experimental approaches and theoretical calculations.^1,12–14,16–27^ Several reaction mechanisms have been proposed, ranging from gas-phase oxidation by atmospheric radicals (Criegee intermediate^16,17,25–27^ and hydroxyl^4,13,14^ and hydroperoxy radicals^18^) to aqueous-phase reaction by O_3_,^20,24,28^ hydrogen peroxide,^29^ and others.^30^ To date, a consensus has emerged that the production of gaseous sulfate is determined by the SO_2_ + OH reaction.^30^ However, recent studies have shown that the formation of sulfate from traditional air quality models does not account for the high sulfate levels observed; this suggests the existence of missing pathways for sulfate production.^31,32^ Thus, new reaction pathways should be explored for the formation of atmospheric sulfate.

New models have been recently proposed for the formation of sulfate in the troposphere, clouds, aerosols, acid rain, and fog to bridge the gap between the modeled and observed sulfate.^20,32–35^ SO_2_ can be quickly taken into fog and rain droplets,^36^ which is followed by its liquid phase oxidation by O_3_ and H_2_O_2_.^20,24,28,29,37,38^ Model studies suggest that the oxidation of SO_2_ in the aqueous phase results in more than 80% of the global sulfate production.^39^ In some of these models, the hydrolysis reaction of SO_2_ plays an important role, and bisulfite anion (HSO_3_^−^) serves as a product of hydrolysis, which is one of the dominant sulfur(iv) species.^40^ The extensive vibrational spectroscopy studies conducted by Simon and Waldman revealed the presence of bisulfite anion (HSO_3_^−^), which resulted from the dissolution of SO_2_ in water.^41–45^ In addition, it has been proven that the existence of NH_3_ in hydrated SO_2_ clusters accelerates the production of HSO_3_^−^;^33^ moreover, the rate of oxidation of dissolved SO_2_ by O_3_ to form sulfate is enhanced.^46^ As another product of hydrolysis, sulfurous acid (H_2_SO_3_) was first generated in an experiment conducted by Schwarz *et al.* in 1988 and was considered stable in the gas phase.^47^ Recent studies have shown that SO_2_ can hydrolyze to produce sulfurous acid (H_2_SO_3_) or HSO_3_^−^ in the gas phase in the presence of acid molecules or clusters such as water, hydrated ammonia, and hydrated sulfuric acid clusters.^33,34^ In addition, sulfurous acid and bisulfite were thought to be potential precursors for the formation of atmospheric aerosols. However, to the best of our knowledge, no studies have been reported on the atmospheric oxidation of H_2_SO_3_/HSO_3_^−^ to produce H_2_SO_4_/HSO_4_^−^.

O_3_ is a reactive oxidant in natural and polluted tropospheres.^48^ It also acts as an oxidant in the aqueous oxidation of SO_2_ (ref. 24) together with transition metal ion catalysts (Co^2+^, Fe^3+^, and Mn^2+^).^20,40^ Furthermore, the gas-phase oxidation of SO_2_ by O_3_ is known to have minor contributions to atmospheric sulfate formation due to its relatively high energy barrier and small rate constant.^49^ However, Cheng *et al.* have reported that the concentrations of O_3_ drop dramatically during the haze period together with an increase in sulfate production; this suggests new possible pathways for the formation of sulfate in the presence of O_3_.^32^ Consequently, we carried out a detailed theoretical investigation on the reaction mechanism of H_2_SO_3_/HSO_3_^−^ + O_3_. To further evaluate the atmospheric implications of the title reactions, the rate constants were calculated utilizing the transition state theory. Our results will provide potential insights into the new mechanisms for atmospheric sulfate generation.

## 2. Computational methods

The structures of the reactants, intermediates, transition states, and products were optimized by the M06-2X functional (density functional theory)^50^ combined with the 6-311++G(3df,3pd) basis set using the Gaussian 09 program.^51^ The M06-2X method has been widely used in the computation of atmospheric reactions and proven to provide reliable results.^52–56^ Harmonic vibrational analyses were conducted to provide the zero-point energy (ZPE) corrections as well as to confirm the minima character of the obtained geometries at the same level of theory (*i.e.* the local minimal with positive frequencies and saddle points with only one imaginary frequency). The ZPE corrections were included in the determination of relative energy for each stationary point. Intrinsic reaction coordinate (IRC) calculations were performed to confirm that the transition states corresponded to the designated reactants and products. To obtain more accurate energetic information, single point energy calculations were carried out at the CCSD(T)/aug-cc-pVTZ level of theory.

Due to the partial biradical character of O_3_,^57^ the reliability of the results obtained had to be determined from the single-reference-based coupled-cluster wave function (CCSD(T)); one of the best methods to determine this is through the *T*_1_ diagnostic.^58^

1 *T*_1_ = ‖*t*_1_‖/(*N*)_elec_^1/2^

where ‖*t*_1_‖ is the Euclidean norm of the *t*_1_ vector of the coupled-cluster wave function, and it is divided by the square root of the number of correlated electrons to normalize *T*_1_. It is recommended that *T*_1_ diagnostic values below 0.045 are acceptable.^59^ All the values of *T*_1_ typically cover the range of 0.0015–0.035 (except for TS16-P), and most of them are lower than 0.025 (see Table S1†). This means that almost all the stationary points have very small multi-reference character, and CCSD(T)/aug-cc-pVTZ could sufficiently describe the electronic states and provide reasonable energies.^23^ For TS16-P, the *T*_1_ diagnostic value is 0.068, indicating that the single-reference-based coupled-cluster method is inadequate to calculate the energies and the multi-reference method is needed to provide a more reliable energy barrier of the transition state TS16-P. Therefore, the complete active space self-consistent field (CASSCF) theory (multi-reference method) was chosen to obtain the reference wave functions of In16 and TS16-P.^60^ Taking into account the dynamic correlation effects, the single point energies of these two stationary points were further refined using the second-order multi-configuration CASPT2 method. The CASPT2//CASSCF calculations were performed using the MOLPRO quantum chemistry package.^61^

## 3. Results and discussion

### 3.1 Mechanism of the H_2_SO_3_ + O_3_ reaction

**Fig. 1:**
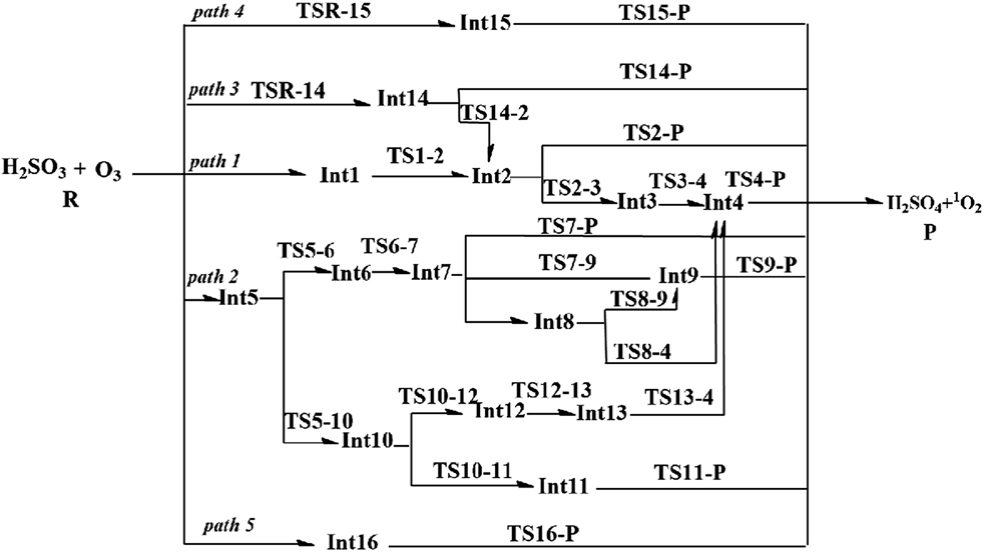
Possible reaction pathways for the H_2_SO_3_ + O_3_ reaction.

**Fig. 2:**
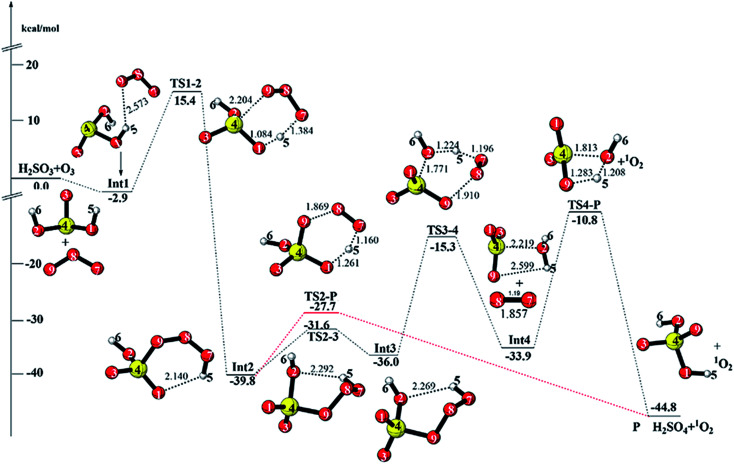
CCSD(T)/aug-cc-pVTZ//M06-2X/6-311++G(3df,3pd) + ZPE energy profile and the optimized geometries of the stationary points for the reaction of H_2_SO_3_ + O_3_ (distances are in angstroms).

**Fig. 3:**
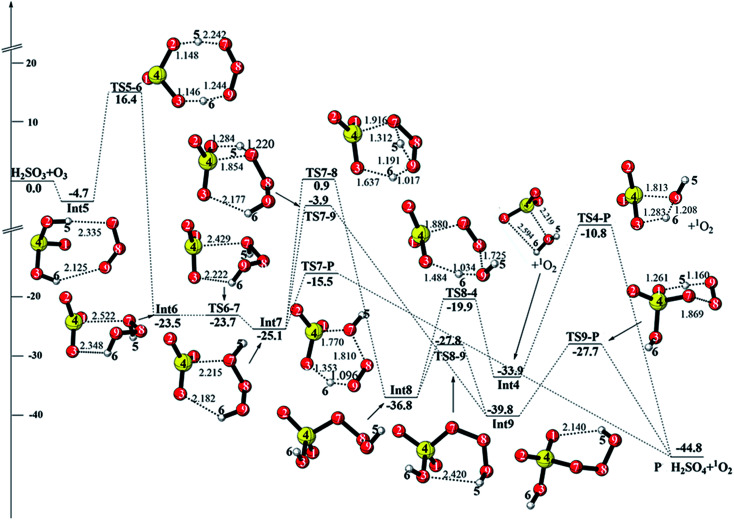
CCSD(T)/aug-cc-pVTZ//M06-2X/6-311++G(3df,3pd) + ZPE energy profile and the optimized geometries of the stationary points for the reaction of H_2_SO_3_ + O_3_ (distances are in angstroms).

**Fig. 4:**
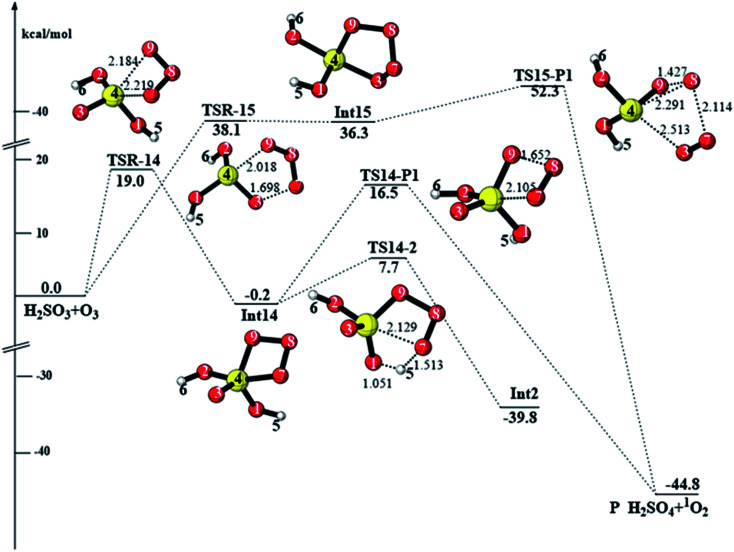
CCSD(T)/aug-cc-pVTZ//M06-2X/6-311++G(3df,3pd) + ZPE energy profile and the optimized geometries of the stationary points for the reaction of H_2_SO_3_ + O_3_ (distances are in angstroms).

Due to the multiple reaction sites in H_2_SO_3_ and O_3_, there are five reaction pathways (paths 1–5) according to the different entrance channels. As shown in Fig. 1, in path 1, path 2, and path 5, the reactions start with the collision of the reactants; this leads to the formation of the hydrogen-bond intermediates Int1, Int5, and Int16, respectively. In paths 3 and 4, the reactions initiate from the cycloaddition reaction processes. The detailed reaction pathways with lower energy barriers as well as the geometrical structures of the stationary points are displayed in Fig. 2–4. The other reaction pathways with much higher energy barriers are presented in the ESI (Fig. S1 and S2†).

#### 3.1.1 Reaction starting from Int1 (path 1)

For path 1, the reaction is initiated from the intermediate Int1 formed by one of the terminal oxygen atoms (O9) in O_3_ approaching one hydrogen atom (H5) in H_2_SO_3_, with a hydrogen bond (O9⋯H5) distance of 2.573 Å. This weak intermolecular hydrogen bond leads to an energy decrease of 2.9 kcal mol^−1^ relative to that of the initial reactants. Subsequently, the reaction proceeds to produce the intermediate Int2*via* the concerted transition state TS1-2 with the energy barrier of 18.3 kcal mol^−1^. In TS1-2, one terminal oxygen atom (O9) in O_3_ adds to sulfur atom, and the other terminal oxygen atom (O7) of O_3_ abstracts H5 atom from H_2_SO_3_ simultaneously. The intramolecular hydrogen bond (O1⋯H5) leads to the six-membered ring structure of Int2, which is −39.8 kcal mol^−1^ lower than the reactants. From Int2, there are two possible reaction pathways, as shown in Fig. 2. For one pathway, Int2 decomposes directly to generate the final products P (H_2_SO_4_ and singlet O_2_) through the transition state TS2-P, in which cleavage of the O8–O9 bond and transfer of H5 from O7 to O1 take place simultaneously with an energy barrier of 12.1 kcal mol^−1^. The large energy release (44.8 kcal mol^−1^) from the initial reactants to the products indicates the thermodynamic feasibility of the H_2_SO_3_ + O_3_ reaction.

For the second pathway, Int2 undergoes an isomerization process to form Int3*via* the rotation of the dihedral angle O8–O9–S–O2 (TS2-3), and the energy barrier is 8.2 kcal mol^−1^. This rotation of the dihedral angle leads to distortion of the six-membered ring; accordingly, Int3 is less stable than Int2 by 3.8 kcal mol^−1^. Then, Int3 decomposes to produce mono-hydrated sulfur trioxide and ^1^O_2_*via* the transition state TS3-4, in which the cleavage of O8–O9 bond and transfer of H5 from O7 to O2 take place simultaneously. The energy barrier of TS3-4 is 20.7 kcal mol^−1^. The hydrated sulfur trioxide can hydrolyze to form sulfuric acid *via* a four-membered proton transfer transition state (TS4-P) with an energy barrier of 23.1 kcal mol^−1^. Although the energy barrier of the SO_3_ hydrolysis reaction is slightly high, this reaction has been proven to be a catalytic reaction, and the energy barrier becomes evidently low or nearly disappears when water molecules or atmospheric acids act as catalysts in the hydrolysis reaction.^62^ Therefore, this reaction pathway will be increasingly important together with the environmental conditions such as high humidity or haze. Thus, the oxidation of H_2_SO_3_ by O_3_ starting from Int1 mainly occurs *via* the pathway reactants → Int1 → Int2 → P with the overall energy barrier of 18.3 kcal mol^−1^.

#### 3.1.2 Reaction initiating from Int5 (path 2)

When two terminal oxygen atoms of O_3_ approach two hydrogen atoms of H_2_SO_3_, the hydrogen-bonded intermediate Int5 forms, with the hydrogen bond lengths of 2.335 and 2.125 Å. Int5 is lower in energy by 4.7 kcal mol^−1^ relative to the reactants. As shown in Fig. 1, there are two channels followed by Int5. We have only discussed the more feasible reactions depicted in Fig. 3. Int5 can isomerize to produce Int6*via* the double H-abstraction transition state TS5-6. This process needs to overcome an energy barrier of 21.1 kcal mol^−1^. In Int6, hydrogen trioxide (H_2_O_3_) interacts with SO_3_ by an intermolecular van der Waals interaction (2.552 Å) and a hydrogen bond (2.348 Å). The torsion of the O7–O8–O9–H6 dihedral angle in Int6 leads to the intermediate Int7*via* the barrierless transition state TS6-7. There are three channels followed by Int7. First, an analogous hydrolysis reaction takes place to produce P*via* the transition state TS7-P. In TS7-P, the proton transfers from O9 to O3, O7 adds to an S atom, and simultaneously, the O7–O8 bond is broken. The energy barrier and energy release of this elementary reaction is 9.6 and 19.7 kcal mol^−1^, respectively.

With respect to the second reaction channels, Int7 undergoes a rearrangement process to produce Int8, which needs to pass through the transition state TS7-8 with an energy barrier of 26.0 kcal mol^−1^. In TS7-8, H5 transfers from O7 to O9, H6 transfers from O9 to O3, and simultaneously, O7 adds to the S atom. Once Int8 is formed, there are two possible pathways connecting to P. For one pathway, Int8 undergoes an isomerization process (TS8-9), yielding the intermediate Int9. The energy barrier of this isomerization process is 9.0 kcal mol^−1^. Then, proton transfer and O7–O8 bond cleavage take place simultaneously *via* the transition state TS9-P to generate P. Otherwise, Int8 can connect to Int4*via* the concerted transition state TS8-4 with an energy barrier of 16.9 kcal mol^−1^, and the following reaction pathways are the same as those in path 1. With regard to the third reaction channel initiating from Int7, it proceeds to produce Int9*via* the transition state TS7-9. The computed energy barrier of TS7-9 is 21.2 kcal mol^−1^.

On the other hand, Int5 can also isomerize to produce Int10*via* the seven-membered ring transition state TS5-10. Following Int10, the reaction proceeds to produce Int11, which finally decomposes to produce H_2_SO_4_ + ^1^O_2_*via*TS11-P (see Fig. S1†). The energy barrier of this reaction path is as high as 35.7 kcal mol^−1^. In addition, Int10 can connect to Int4*via* a continuous isomerization reaction process. These reaction pathways are not competitive due to their high energy barriers, and the details of these reaction pathways are presented in the ESI (see Fig. S1†). To summarize, among all the pathways discussed in this section, the reaction path: reactants → Int5 → Int6 → Int7 → P is the most favorable with the total energy barrier of 21.1 kcal mol^−1^.

#### 3.1.3 Reactions initiating from Int14, Int15, and Int16 (path 3, path 4, and path 5)

When two terminal oxygen atoms in O_3_ add to the S atom, the cycloaddition product Int14 is formed. The energy barrier (TSR-14) of this cycloaddition process is 19.0 kcal mol^−1^. As shown in Fig. 4, there are two further reaction channels initiating from Int14. For the first reaction channel, Int14 decomposes to the products *via* the transition state TS14-P with the rupture of the O7–S and O8–O9 bonds. The energy barrier of this transition state is 16.7 kcal mol^−1^. For the second reaction channel, Int14 connects to Int2*via* the transition state TS14-2, in which the S–O7 bond ruptures, and simultaneously, H5 transfers from O1 to O7. This process needs to pass through an energy barrier of 7.9 kcal mol^−1^. Followed by Int2, the reaction is the same as that in path 1.

The terminal oxygen atoms in O_3_ can also add to the S=O bond; this yields the five-membered structure Int15 (path 4). Then, Int15 dissociates to produce P*via*TSR-15, with an energy barrier (TSR-15) of 52.3 kcal mol^−1^. Thus, this reaction channel is not competitive.

For path 5, the reaction initiates from the hydrogen-bonded intermediate Int16, which is followed by oxygen addition *via* the transition state TS16-P to produce H_2_SO_4_ + ^1^O_2_. The energy barrier of TS16-P is 29.1 kcal mol^−1^ at the CCSD(T)/aug-cc-pVTZ level. However, the *T*_1_ diagnostic value of TS16-P is 0.068. Therefore, the CASPT2//CASSCF/aug-cc-pVTZ method was employed to further verify the energy barrier of TS16-P, and the computed energy barrier of TS16-P was 44.1 kcal mol^−1^ (see Fig. S2†). This further confirms that this reaction channel is kinetically unfavorable. To conclude, among the reactions discussed in this section, the reaction pathway reactants → Int14 → P is the most feasible with the total energy barrier of 19.0 kcal mol^−1^.

### 3.2 Mechanism of the HSO_3_^−^ + O_3_ reaction

**Fig. 5:**
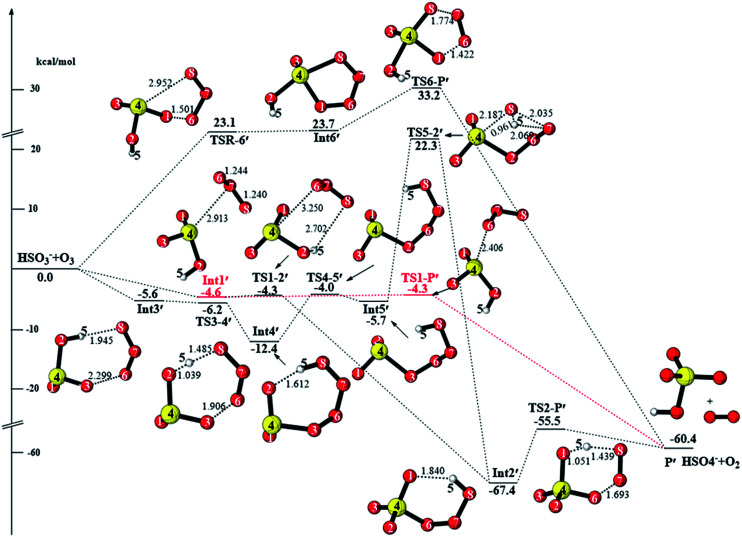
CCSD(T)/aug-cc-pVTZ//M06-2X/6-311++G(3df,3pd) + ZPE energy profile and the optimized geometries of the stationary points for the reaction of HSO_3_^−^ + O_3_ (distances are in angstroms).

As discussed in the introduction, SO_2_ can easily hydrolyze to produce bisulfite in the gas phase, and the detailed mechanisms for the oxidation of HSO_3_^−^ by O_3_ are presented in Fig. 5. The intramolecular interaction of HSO_3_^−^ and O_3_ leads to the formation of the intermediate Int1′ with the binding energy of 4.6 kcal mol^−1^ and then Int1′ decomposes to the bisulfate ion (HSO_4_^−^) and ^1^O_2_ products *via* the transition state TS1-P′. In TS1-P′, O6 adds to the sulfur atom, and simultaneously, the O6–O7 bond breaks. The barrier height of this process is only 0.3 kcal mol^−1^. The reaction is exothermic by as much as 60.4 kcal mol^−1^.

The intermediate Int1′ can connect to Int2′*via* the transition state TS1-2′. Similar to TS1-2, H5-abstraction and oxygen addition take place simultaneously in TS1-2′. The energy barrier of TS1-2′ is 0.3 kcal mol^−1^ relative to that of Int1′. Then, the reaction proceeds by the concerted transfer of H5 from O8 to O1 and O6–O7 bond cleavage *via* the transition state TS2-P′ to produce the terminal products **P′** (HSO_4_^−^ + ^1^O_2_). The energy barrier of TS2-P′ is 11.9 kcal mol^−1^.

In addition, the cycloaddition reaction can take place either by the transition state TSR-6′ or TS3-4′ to form the five-membered ring intermediate Int6′ or seven-membered ring intermediate Int4′, respectively. These two cycloaddition reactions are similar to the reaction processes *via* the transition states TSR-15 and TS5-10 (Fig. S2†). Int6′ decomposes into HSO_4_^−^ and ^1^O_2_ by O–O bond cleavage *via* the transition state TS6-P′. Int4′ can connect to Int2′*via* a continuous isomerization reaction *via*TS4-5′ and TS5-2′ and then decompose to HSO_4_^−^ and ^1^O_2_*via* the transition state TS2-P′. However, the two reaction pathways discussed in this section are not competitive due to their relatively high energy barriers (23.1 and 28.0 kcal mol^−1^). Therefore, among the reactions of HSO_3_^−^ + O_3_, the reaction pathway reactant (HSO_3_^−^ + O_3_) → Int1′ → TS1-P′ → P′(HSO_4_^−^ + ^1^O_2_) is the most feasible with the total energy barrier of only 0.3 kcal mol^−1^.

### 3.3 Comparison of the molecular orbitals of the intermediates

**Fig. 6:**
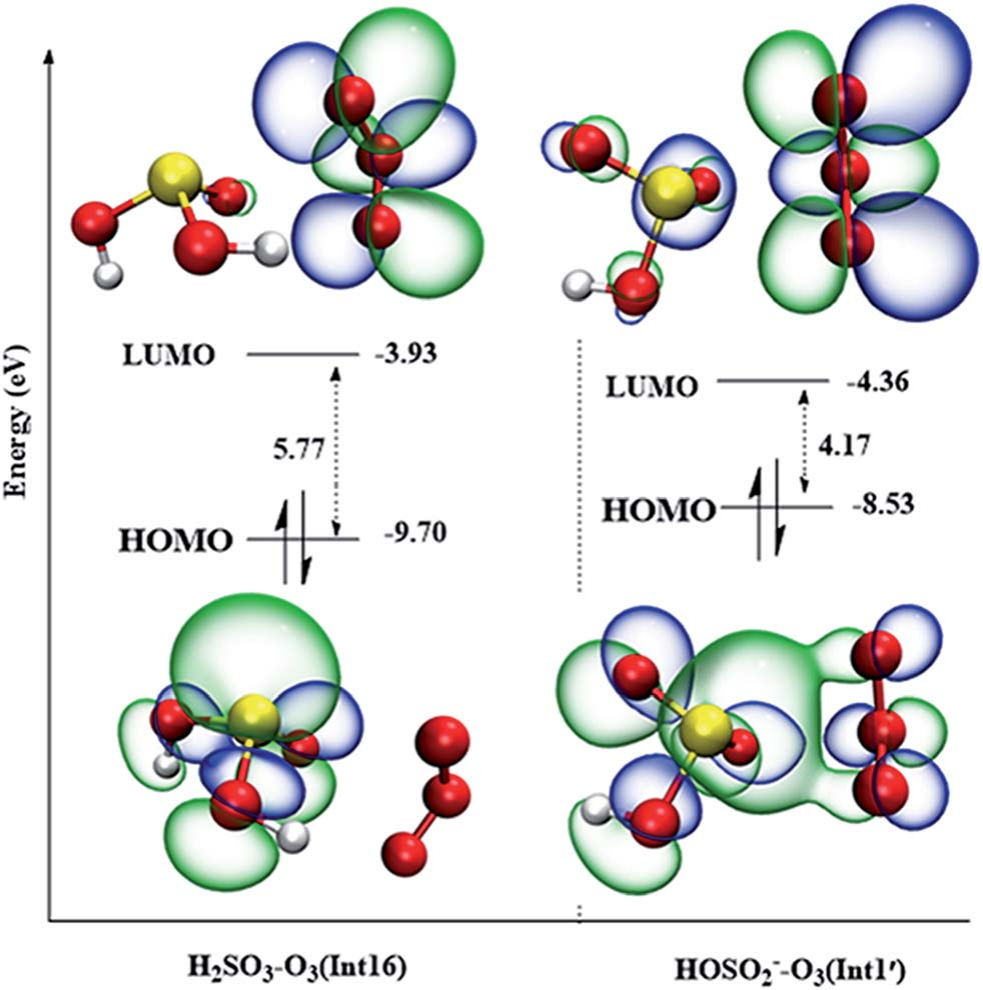
HOMOs and LUMOs of Int1′ (HSO_3_^−^–O_3_) and Int16 (H_2_SO_3_–O_3_) (isosurfaces 0.03 e/Å^3^).

When a H_2_SO_3_ molecule dissociates one proton forming the HSO_3_^−^ anion, the reaction mechanisms are entirely different. Oxygen addition from Int1′ in the HSO_3_^−^ + O_3_ reaction is the most favorable reaction pathway with an energy barrier of only 0.3 kcal mol^−1^ (Fig. 5), whereas the similar reaction path from Int16 in the H_2_SO_3_ + O_3_ reaction is unfeasible due to is high energy barrier of over 29 kcal mol^−1^ (see Fig. S2†). To further explain the huge differences in the energy barriers of these two reaction channels, the molecular orbital analysis is a good choice. It is generally recognized that the kinetic stability of reactants or reactant intermediates can be quantitatively determined using HOMO–LUMO energy separation based on the simple Hückel theory.^63^ The smaller the HOMO–LUMO gap, the more energetically favorable the electrons to add to a high-lying LUMO from a low-lying HOMO. The highest occupied molecular orbital (HOMO) and lowest unoccupied molecular orbital (LUMO) of the intermediates Int1′ and Int16 are drawn in Fig. 6.

As can be seen from Fig. 6, for both Int1′ and Int16, their HOMOs are mainly occupied on the sulfur atom, and the LUMOs are mainly distributed in the anti-bonding π* orbital of O_3_. The HOMO–LUMO gap in Int1′ (4.17 eV) is smaller than that in Int16 (5.77 eV); this indicates that the electrons transfer easily from the HOMO orbital to the LUMO orbital in Int1′. Furthermore, there are some electron distributions in the π*(O–O) orbital of O_3_ in Int1′ as well as some overlap between the orbital of lone pair electrons in the sulfur atom and the π*(O–O) anti-bonding orbital of O_3_. The electron distributions in π*(O–O) lead to an increase in the electron population of π*(O–O); this will ultimately facilitate O–O bond activation. The O–O bond activation can also be reflected by the different O–O bond lengths in Int1′ and Int16. As shown in Fig. 6 and S2,† the O–O bond lengths of O_3_ in Int1′ are 1.244 and 1.240 Å, which are larger than those in Int16 (1.227 and 1.229 Å, respectively). Therefore, the smaller HOMO–LUMO gap combined with the electron distributions of π*(O–O) in O_3_ may be one reason for the lower energy barrier of TS1-P′ in the HSO_3_^−^ + O_3_ reaction.

### 3.4 Kinetics and implication in atmospheric chemistry

The rate constants for the main pathways of the H_2_SO_3_/HSO_3_^−^ + O_3_ reactions were calculated in terms of the transition state theory (TST) with the Wigner tunneling correction. There are two competitive reaction pathways for the H_2_SO_3_ + O_3_ reaction. The reaction pathway reactants → Int1 → Int2 → P with the total energy barrier of 18.3 kcal mol^−1^ is the most favorable reaction pathway. The reaction pathway reactants → Int14 → P may be a competitive reaction path with the total energy barrier of 19.0 kcal mol^−1^. Therefore, both reaction pathways were considered. The reaction pathways can be depicted as follows, and the computational details are presented in the ESI.†R1
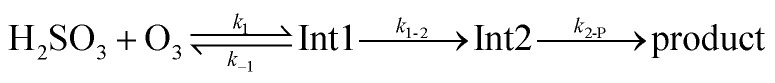
R2
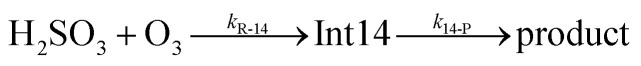
R3
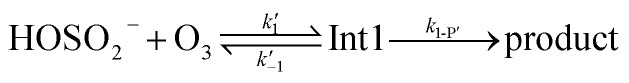


Considering the atmospheric temperature range from 212 to 298 K in the troposphere and stratosphere of the Earth, since the altitude changes from 0 km to 50 km,^62^ the rate constants have been calculated in the temperature range of 200–320 K. The calculated rate constants are presented in Table 1. For the reaction R1, the rate constants stay within the range of 3.94 × 10^−31^–8.68 × 10^−25^ cm^3^ per molecule per s. The rate constants of reaction R2 are about 4–5 orders of magnitude smaller than those of reaction R1; this highlights that the oxidation of H_2_SO_3_ by O_3_ takes place mainly *via* reaction R1 within the studied temperature range. The rate constants of R3 are in the range of 8.89 × 10^−11^–1.08 × 10^−10^ cm^3^ per molecule per s, which are 15–19 orders of magnitude larger than those of reaction R1. Vahedpour *et al.*^49^ previously reported the reaction mechanism and kinetics of the SO_2_ + O_3_ reaction. The rate constant of SO_2_ + O_3_ is 2.30 × 10^−23^ cm^3^ per molecule per s at room temperature, which is approximately 13 orders of magnitude smaller than that of reaction R3. Although SO_3_ produced from the SO_2_ + O_3_ reaction can further transform into H_2_SO_4_*via* the atmospheric hydrolysis reaction, this reaction plays a minor role in atmospheric sulfate formation. Conversely, SO_2_ may first hydrolyze to form sulfite in the atmosphere; then, it is easily oxidized to produce sulfate in the presence of O_3_. This means that sulfite may be a key intermediate in the atmospheric production of sulfate, and O_3_ is a potentially important oxidant besides OH for atmospheric sulfate formation.

**Table 1:** Values of the equilibrium constants (*K*_eq_, in molecules per cm), tunneling factor (*κ*), collision rate (*k*_1_, in cm^3^ per molecule per s^−1^), unimolecular rate constant (*k*_TS_, s^−1^), and overall rate constant (*k*_tot_, in cm^3^ per molecule per s) for the reactions of H_2_SO_3_/HSO_3_^−^ + O_3_

Reaction	*T*/K	200	220	240	260	280	298	300	320
H_2_SO_3_ + O_3_ (R1)	*K*_eq1_	1.37 × 10^−22^	8.55 × 10−^23^	5.92 × 10^−23^	4.44 × 10^−23^	3.54 × 10^−23^	3.01 × 10^−23^	2.96 × 10^−23^	2.58 × 10^−23^
	*k*_TS1-2_	2.87 × 10^−9^	1.51 × 10^−7^	4.08 × 10^−6^	6.59 × 10^−5^	7.12 × 10^−4^	4.60 × 10^−3^	5.58 × 10^−3^	3.37 × 10^−2^
	*κ*(*k*_TS1-2_)	1.84	1.70	1.58	1.50	1.43	1.38	1.37	1.33
	*k*_TS2-P_	6.56 × 10^−1^	9.92 × 10^0^	9.55 × 10^1^	6.49 × 10^2^	3.36 × 10^3^	1.22 × 10^4^	1.40 × 10^4^	4.88 × 10^4^
	*κ*(*k*_TS2-P_)	3.18	2.80	2.51	2.29	2.11	1.98	1.97	1.85
	*k*_uni_	2.87 × 10^−9^	1.51 × 10^−7^	4.08 × 10^−6^	6.59 × 10^−5^	7.12 × 10^−4^	4.60 × 10^−3^	5.58 × 10^−3^	3.37 × 10^−2^
	*k*	3.94 × 10^−31^	1.29 × 10^−29^	2.41 × 10^−28^	2.93 × 10^−27^	2.52 × 10^−26^	1.38 × 10^−25^	1.65 × 10^−25^	8.68 × 10^−25^
H_2_SO_3_ + O_3_ (R2)	*k*_TSR-14_	7.13 × 10^−37^	5.44 × 10^−35^	2.05 × 10^−33^	4.47 × 10^−32^	6.37 × 10^−31^	5.17 × 10^−30^	6.43 × 10^−30^	4.92 × 10^−29^
	*κ*(*k*_TSR-14_)	1.24	1.20	1.17	1.14	1.12	1.11	1.11	1.09
	*k*_TS14-P_	6.42 × 10^−6^	3.03 × 10^−4^	7.62 × 10^−3^	1.18 × 10^−1^	1.24 × 10^0^	7.96 × 10^0^	9.65 × 10^0^	5.84 × 10^1^
	*κ*(*k*_TS14-P_)	2.23	2.01	1.85	1.73	1.63	1.55	1.55	1.48
	*k*	7.13 × 10^−37^	5.44 × 10^−35^	2.05 × 10^−33^	4.47 × 10^−32^	6.37 × 10^−31^	5.17 × 10^−30^	6.43 × 10^−30^	4.92 × 10^−29^
HSO_3_^−^ + O_3_ (R3)	*K*′_eq_	5.37 × 10^−20^	2.22 × 10^−20^	1.09 × 10^−20^	6.10 × 10^−21^	3.78 × 10^−21^	2.64 × 10^−21^	2.54 × 10^−21^	1.82 × 10^−21^
	*K*′_1_	8.91 × 10^−11^	9.76 × 10^−11^	9.34 × 10^−11^	1.02 × 10^−10^	1.05 × 10^−10^	1.09 × 10^−10^	1.09 × 10^−10^	1.13 × 10^−10^
	*k*′_−1_	1.66 × 10^9^	4.40 × 10^9^	8.58 × 10^9^	1.67 × 10^10^	2.79 × 10^10^	4.12 × 10^10^	4.29 × 10^10^	6.18 × 10^10^
	*k*′_TS1-P_	8.77 × 10^11^	9.65 × 10^11^	1.05 × 10^12^	1.12 × 10^12^	1.18 × 10^12^	1.24 × 10^12^	1.25 × 10^12^	1.30 × 10^12^
	*κ*(*k*′_TS1-P_)	1.01	1.01	1.01	1.01	1.01	1.00	1.00	1.00
	*k*′	8.89 × 10^−11^	9.72 × 10^−11^	9.26 × 10^−11^	1.00 × 10^−10^	1.03 × 10^−10^	1.05 × 10^−10^	1.05 × 10^−10^	1.08 × 10^−10^

Speculating that this oxidation reaction is responsible for the atmospheric removal of sulfite species, the atmospheric lifetime (*τ*) of sulfite can be estimated by the expression *τ* = (*k*′_tot_ × [O_3_])^−1^ according to the reaction of HSO_3_^−^ + O_3_. When the O_3_ average concentration is 7.0 × 10^11^ molecule per cm^−3^ in the atmosphere,^64^ the lifetime of sulfite is estimated to be 0.014 s at 298 K. This result indicates that once the atmospheric sulfur dioxide transforms into sulfite species, it can be oxidized to sulfate immediately. It has been reported that the production of sulfite from SO_2_ is almost a barrierless process in atmospheric aerosols in the presence of water, ammonia or atmospheric acids.^33,34^ Consequently, the oxidation of sulfite by O_3_ may provide a new reaction mechanism for the missing source of atmospheric sulfate.

It is worth mentioning that the concentration of O_3_ is quite different in the stratosphere. The ozonosphere exits at an altitude of 20–25 km in the stratosphere, where the O_3_ concentration reaches its maximum (∼2.7 × 10^14^ molecule per cm^−3^).^65,66^ Under this condition, the lifetime of sulfite is estimated to be approximately 4.0 × 10^−3^ s. Thus, sulfite pollutants reaching the stratosphere may contribute to the destruction of the ozone layer.

## 4. Conclusion

The reaction mechanism and kinetic investigations on the reaction of H_2_SO_3_/HSO_3_^−^ + O_3_ were performed theoretically. According to our results, it is clear that the oxidation of H_2_SO_3_ by O_3_ plays a minor role in the formation of sulfuric acid, and the energy barrier of the favorable reaction channel is 18.3 kcal mol^−1^. For the oxidation reaction of HSO_3_^−^ + O_3_, the energy barrier of the most feasible pathway is only 0.3 kcal mol^−1^. In addition, the kinetic analysis verified that the oxidation of HSO_3_^−^ played an important role in the formation of atmospheric sulfate. Accordingly, in this study, the reaction mechanism of the atmospheric oxidation of tetravalent sulfur species (sulfurous acid/sulfite) to produce hexavalent sulfur species (sulfuric acid/sulfate) was proposed, which might be responsible for the missing source of sulfate and particulate matter in the atmosphere. In the stratosphere with a high concentration of O_3_, the lifetime of HSO_3_^−^ decreases to 4.0 × 10^−3^ s; this means that the atmospheric sulfite species may have a potential effect on the destruction of the ozone layer. Our results predict that the sulfite species may be important intermediates in the oxidation of sulfur dioxide to produce sulfate, and O_3_ is an important potential oxidant besides OH for the formation of atmospheric sulfate.

## Conflicts of interest

There are no conflicts of interest to declare.

## Supplementary Material

RA-008-C8RA00411K-s001
