# Supplementary material for: Theoretical study of the oxidation reactions of sulfurous acid/sulfite with ozone to produce sulfuric acid/sulfate with atmospheric implications
Source: RSC Adv. 2018 Feb 20;8(15):7988–96. doi: 10.1039/c8ra00411k (PMC9078479; doi:10.1039/c8ra00411k)
Supplement: RA-008-C8RA00411K-s001 [file RA-008-C8RA00411K-s001.pdf]

Electronic Supplementary Material (ESI) for RSC Advances.
This journal is © The Royal Society of Chemistry 2018

Supporing Information

Theoretical Study of Oxidation Reactions of Sulfurous Acid/Sulfite with Ozone to Produce Sulfuric Acid/Sulfate with Atmospheric Implications

Fang Sheng[1], Liu Jingjing[2*], Chen Yu[1], Fu-Ming Tao[3], Duan XueMei[1*], Liu Jing-yao[1]

[1]Institute of Theoretical Chemistry, Jilin University, Changchun 130023, China

[2]Department of Chemistry and Key Laboratory of Organic Optoelectronics & Molecular Engineering of Ministry of Education, Tsinghua University, Beijing 100084, China.

[3]Department of Chemistry and Biochemistry, California State University, Fullerton, California 92834, United States

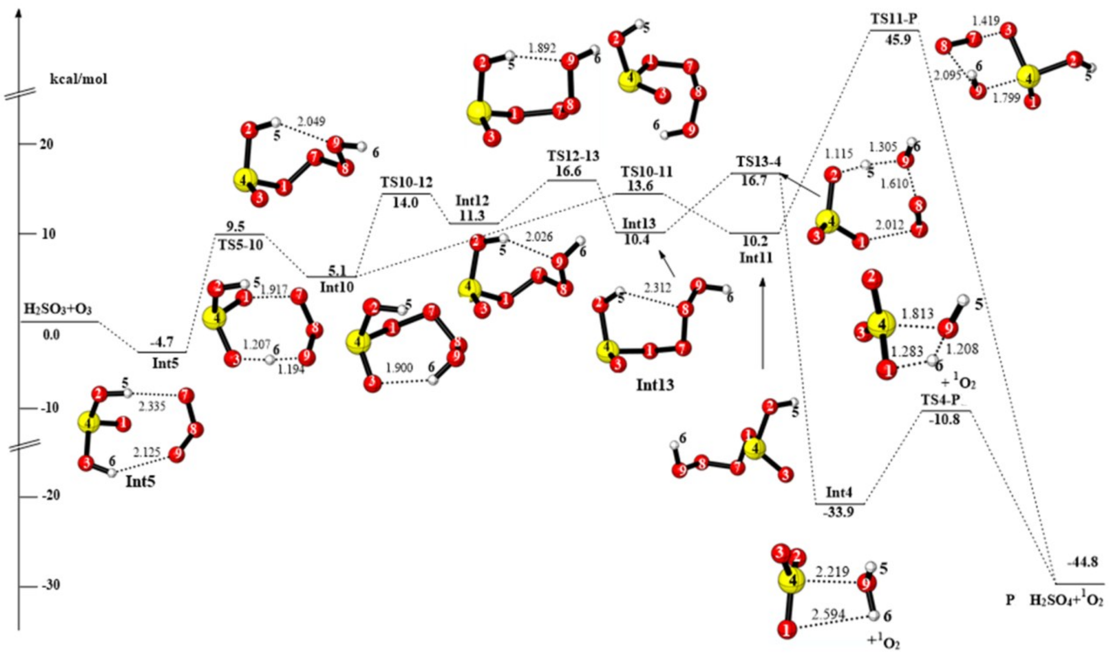

Fig. S1 CCSD(T)/aug-cc-pVTZ // M06-2X /6-311++G(3df,3pd) energy profile and the optimized geometries of the stationary points for the reaction of H_2SO_3 + O_3 (distances are in angstroms)

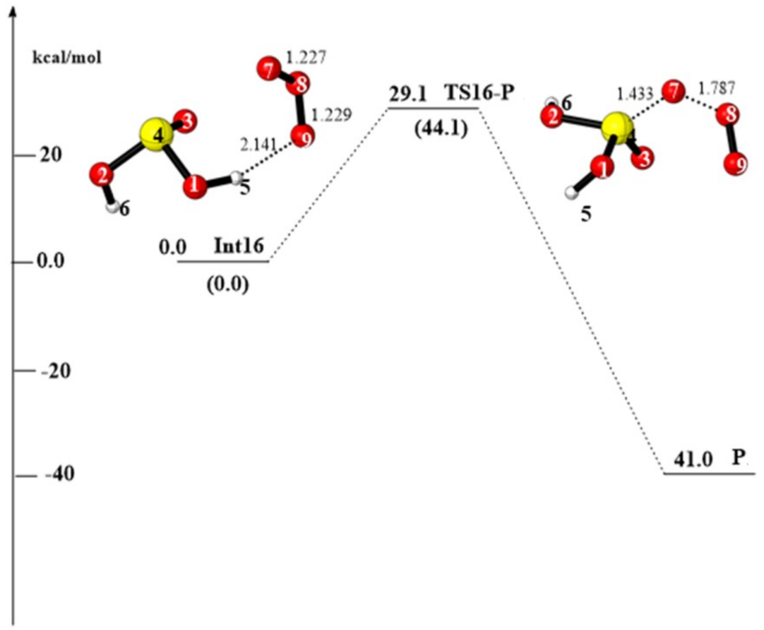

Figure S2 CCSD(T)/aug-cc-pVTZ // M06-2X /6-311++G(3df,3pd) + ZPE energy profile and the optimized geometries of the stationary points for the reaction of H_2SO_3 + O_3 . The data in brackets are calculated at CASPT2//CASSCF/ aug-cc-pVTZ level of theory. (distances are in angstroms).

Table S1 Molecular orbitals and occupation numbers of Int16 from the CASSCF calculations

Orbital NO.	CAS(12e,12o)	Occ.	Orbital NO.	CAS(12 e,12o)	Occ.
HOMO	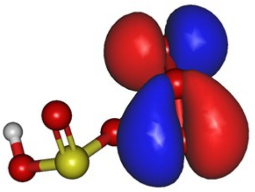	1.78	LOMO	0.22	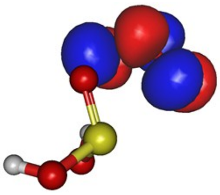
HOMO-1	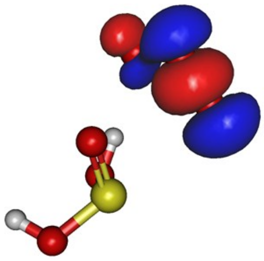	1.97	LOMO -1	0.33	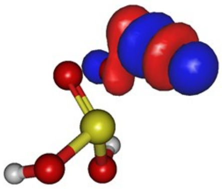
HOMO-2	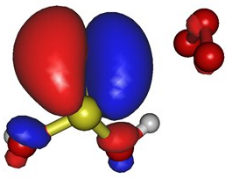	1.97	LOMO -2	0.029	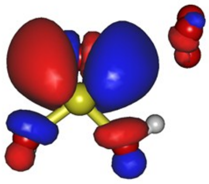
HOMO-3	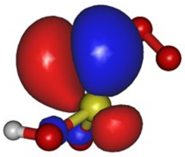	1.97	LOMO-3	0025	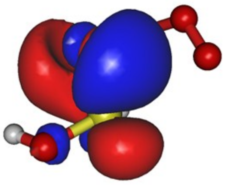
HOMO-4	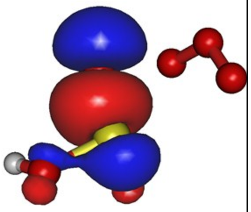	1.97	LOMO-4	0.024	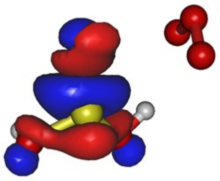
HOMO-5	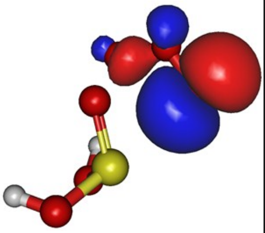	1.98	LOMO-5	0.015	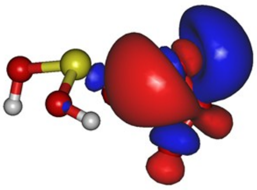

Table S2 Molecular orbitals and occupation numbers of TS16-P from the CASSCF calculations.

**Orbital NO.**	**CAS(12e,12o)**	**Occ.**	**Orbital NO.**	**CAS(12e,12o)**	**Occ.**
HOMO	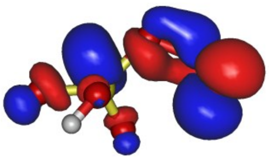	1.37	LOMO	0.63	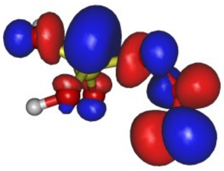
HOMO-1	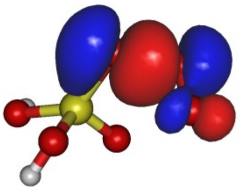	1.94	LOMO -1	0.065	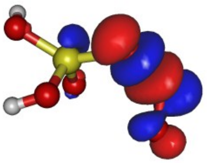
HOMO-2	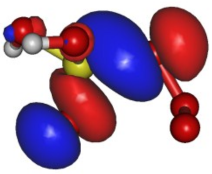	1.96	LOMO -2	0.039	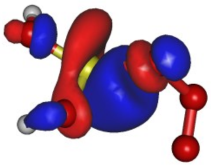
HOMO-3	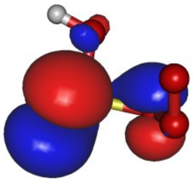	1.97	LOMO-3	0026	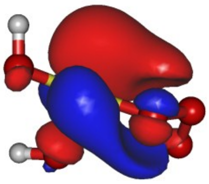
HOMO-4	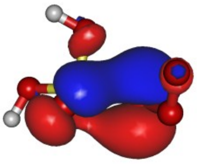	1.98	LOMO-4	0.017	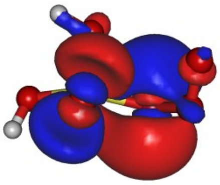
HOMO-5	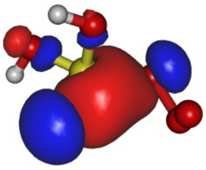	1.98	LOMO-5	0.013	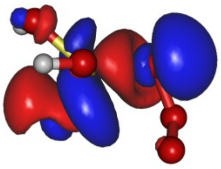

Rate Constant Calculations

For reaction (R1), there are two successive unimolecular reaction steps from Int1, and the total unimolecular rate constants can be calculated according to canonical unified statistical theory as expressed by eq 1:

$$\frac{1}{\mathrm{k}_{uni}} = \frac{1}{k_{1-2}} + \frac{1}{k_{2-P}} \qquad (1)$$

When applying the steady-state approximation for Int1, and assuming that Int1 is in equilibrium with the initial reactants, the total rate constants can be obtained from

$$\mathrm{k} = \frac{k_1 k_{uni}}{k_{-1} + k_{uni}} \qquad (2)$$

When $k_{uni} \ll k_{-1}$, the total rate constants can be obtained from eq 3:

$$k = K_{eq} k_{uni} \qquad (3)$$

where K_{eq} is the equilibrium constant for the formation of Int1 and was calculated using the MultiWell software.[1] k_{uni} were carried out in terms of the transition state theory (TST) with Wigner Tunneling corrections and KiSThelP software[2] was used. With regard to reaction (2), the total rate constants were obtained according to the following eq 4.

$$\frac{1}{\mathrm{k}} = \frac{1}{k_{R-14}} + \frac{1}{k_{14-P}} \qquad (4)$$

For the $HSO_3^- + O_3$ reaction, the energy barrier of TS1-P is merely 0.3 kcal/mol, which indicates that k_{-1}' is not much greater than k_{uni}'. Accordingly, the overall rate constants are evaluated according to eq 5, where k_1' is calculated by hard-sphere collision theory[3] accompanied by obtaining k_{-1} based on the data of K_{eq}' and k_{-1}'.

$$\mathrm{k}' = \frac{k_1' k_{uni}'}{k_{-1}' + k_{uni}'} \qquad (5)$$

1 (a) J. R. Barker, T. L. Nguyen, J. F. Stanton, C. Aieta, M. Ceotto, F. Gabas, T. J. D. Kumar, C. G. L. Li, L. L. Lohr, A. Maranzana, N. F. Ortiz, J. M. Preses, J. M. Simmie, J. A. Sonk, and P. J. Stimac; MultiWell-2017 Software Suite; J. R. Barker, University of Michigan, Ann Arbor, Michigan,USA,2016; http://clasp-research.engin.umich.edu/

multiwell/. (b) John R. Barker, Int. J. Chem. Kinetics, 33, 232-45 (2001). (c) John R. Barker, Int. J. Chem. Kinetics, 41, 748-763 (2009).".

2 S. Canneaux, F. Bohr and E. Henon, *Journal of Computational Chemistry*, 2014, **35**, 82-93.

3 W. H. Miller, *Journal of Chemical Physics*, 1976, **65**, 2216-2223.

Table S3 The electronic energies, ZPE, and T1 diagnostic values of the stationary points in H_2SO_3+O_3 reaction (Energy unit: Hartree)

Species	E_{elect}, M062x	ZPE	E_{elect}, CCSD(T)	T1 diagnostic value
H_2SO_3	-625.05417	0.03359	-624.33004	0.017
O_3	-225.39663	0.008152	-225.14923	0.024
$^1O_2$	-700.28673	0.040145	-699.46154	0.015
H_2SO_4	-150.25992	0.004046	-150.09154	0.015
Int1	-850.45857	0.043578	-849.48564	0.021
Int2	-850.54957	0.04819	-849.54909	0.017
Int3	-850.54306	0.04805	-849.54288	0.017
Int4	-700.26348	0.03869	-699.44279	0.016
Int5	-850.46197	0.04367	-849.48864	0.020
Int6	-850.51798	0.04660	-849.5216	0.017
Int7	-850.52193	0.04686	-849.52432	0.017
Int8	-850.54428	0.04777	-849.54387	0.017
Int9	-850.54957	0.04819	-849.54909	0.016
Int10	-850.46264	0.04567	-849.47506	0.02
Int11	-850.45279	0.04505	-849.46633	0.019
Int12	-850.45244	0.04546	-849.46499	0.019
Int13	-850.45392	0.04495	-849.46594	0.02
Int14	-850.47947	0.04812	-849.48593	0.016
Int15	-850.40811	0.04587	-849.42550	0.035
Int16	-850.4605	0.04378	-849.42059	0.021
TS1-2	-850.43147	0.04156	-849.45461	0.033
TS2-P	-850.51799	0.04320	-849.52480	0.024
TS2-3	-850.53635	0.04762	-849.53549	0.017
TS3-4	-850.49779	0.04314	-849.50513	0.024
TS4-P	-700.22609	0.03572	-699.40301	0.016
TS5-6	-850.41636	0.03626	-849.44766	0.019
TS6-7	-850.5179	0.04641	-849.52163	0.017
TS7-8	-850.47437	0.04309	-849.47919	0.017
TS7-9	-850.48522	0.04350	-849.48719	0.018
TS7-P	-850.4983	0.04442	-849.50664	0.025
TS8-4	-850.50592	0.04533	-849.51464	0.024
TS8-9	-850.52944	0.04763	-849.52938	0.018
TS9-P	-850.51799	0.04320	-849.52480	0.024
TSR-14	-850.42816	0.04418	-849.45136	0.026
TSR-15	-850.3921	0.04394	-849.42069	0.035

TS15-P	-850.37447	0.04231	-849.39650	0.02
TS14-P	-850.44297	0.04470	-849.45588	0.034
TS14-2	-850.46451	0.04429	-849.47038	0.019
TS5-10	-850.43709	0.04005	-849.46244	0.033
TS10-12	-850.44704	0.04422	-849.45937	0.019
TS12-13	-850.4421	0.04416	-849.45516	0.020
TS10-11	-850.44766	0.044705		0.01891727
TS13-4	-850.40043	0.039992	-849.45088	0.041
TS11-P	-850.35849	0.042508	-849.4069	0.029
TS16-P	-850.39444	0.042045	-849.43925	0.068

Table S4 The electronic energies, ZPE, and T1 diagnostic values of the stationary points in HSO_3^- + O_3 reaction (Energy unit: Hartree)

Species	E_{elect}, M062x	ZPE	E_{elect}, CCSD(T)	T1 diagnostic value
HSO_3^-	-624.52347	0.02158	-623.79903	0.020
HSO_4^-	-699.78183	0.02786	-698.95511	0.016
Int1′	-849.93528	0.03156	-848.95745	0.022
Int2′	-850.06497	0.03648	-849.06245	0.017
Int3′	-849.93339	0.03155	-848.95907	0.028
Int4′	-849.95975	0.03325	-848.97160	0.021
Int5′	-849.94915	0.03305	-848.96074	0.021
Int6′	-849.89721	0.03232	-848.91309	0.026
TS1-2′	-849.93289	0.03103	-848.95694	0.022
TS2-P′	-850.03449	0.03275	-849.03973	0.024
TS1-P′	-849.93416	0.03124	-848.95666	0.025
TS3-4′	-849.93165	0.03071	-848.95920	0.035
TS4-5′	-849.94658	0.03271	-848.95767	0.021
TS5-2′	-849.87216	0.03090	-848.91387	0.031
TSR-6′	-849.89376	0.02983	-848.91156	0.028
TS6-P′	-849.86390	0.03105	-848.89663	0.031

Table S5 The Cartesian coordinates of all species for the H_2SO_3/$HOSO_2^-$ + O_3 reaction. The Cartesian coordinates are obtained at the M06-2X /6-311++G(3df,3pd) level of theory.

H_2SO_3

S	-0.00001700	0.11143200	-0.43456400
O	0.00094900	1.35290300	0.31108300
O	1.23375100	-0.73921700	0.15059400
H	1.46689900	-0.39570600	1.02675500

H	-1.46633200	-0.39528100	1.02766100
O	-1.23473600	-0.73767600	0.15065000

O_3

O	1.05461800	-0.20990200	0.00000000
O	0.00000000	0.41968000	0.00000000
O	-1.05461800	-0.20977800	0.00000000

$^1O_2$

O	0.00000000	0.00000000	0.59284400
O	0.00000000	0.00000000	-0.59284400

H_2SO_4

S	0.00011600	0.09103200	0.12032300
O	0.00187100	1.49456500	0.19786600
O	0.00003800	-0.76026400	1.25899200
H	-1.55790300	-1.17523100	-0.51459600
H	1.55586100	-1.17755000	-0.51523800
O	1.22291500	-0.31245100	-0.78517600
O	-1.22480000	-0.30981600	-0.78359900

Int1

S	-1.26163700	0.11324000	-0.37004300
O	-2.60205800	-0.19713800	0.04177600
O	-0.72600400	1.05951000	0.82761700
H	0.19242600	1.30896900	0.64619200
H	-0.64442100	-1.58760400	0.77550600
O	-0.34947700	-1.18907400	-0.05890500
O	1.74176600	0.66609100	-0.88887300
O	2.33727600	-0.22878600	-0.28527200
O	2.17827100	-0.30225200	0.92603000

Int2

S	-0.71328800	-0.17682100	0.01326600
O	-1.72114800	-0.41910900	0.96918600
O	-0.15320200	-1.17790600	-0.81378600
H	1.96245900	-0.97438700	-0.56818500
H	-1.70880900	1.61014800	-0.48079500
O	-1.14948600	0.97534100	-0.95003300
O	0.46514100	0.50926600	0.85954500
O	1.56859500	0.74576000	-0.01537800
O	2.38497100	-0.35918100	0.05505600

Int3

S	-0.81170400	-0.04065600	-0.00250200
O	-1.12852100	-0.34363600	1.34108100
O	-1.71584800	-0.20749500	-1.06563500
H	2.02604500	1.12353000	-0.18835100
H	-0.37307700	1.82801400	0.84663700
O	-0.21638300	1.42997400	-0.02092700
O	0.48828200	-0.84428700	-0.48131500
O	1.55855000	-0.56835300	0.41831800
O	2.43070600	0.24616600	-0.26880500

Int4

S	0.40805800	-0.00010300	-0.03598900
O	0.37321800	1.22441000	-0.74908400
O	0.75782600	-0.00397400	1.33197000
H	-2.11921900	-0.77271500	-0.18076900
H	-2.12201300	0.77565200	-0.17578300
O	-1.78697000	0.00058100	0.28857800
O	0.36996300	-1.22117900	-0.75491600

Int5

S	1.47768100	0.22536300	0.25864300
O	0.39726800	0.19811100	1.22822300
O	1.54406600	-1.24956200	-0.37056000
H	0.65375900	-1.63436000	-0.39051600
H	-0.06820800	1.03675400	-0.95899700
O	0.90380000	1.04850000	-0.99230600
O	-2.00153100	0.94676500	-0.08112500
O	-2.25256800	-0.21614900	0.21655000
O	-1.61959200	-1.10369000	-0.34938000

Int6

S	-1.09806600	-0.12688100	-0.02752800
O	-1.07405500	-0.85179400	1.18005800
O	-1.70104200	1.15016200	-0.09302500
H	0.98849000	1.61588800	-0.59986900
H	1.65751600	-1.11835600	-0.83467400
O	-0.62402000	-0.72246000	-1.22505800
O	1.92972200	-0.93110400	0.07631400
O	2.26747400	0.40834100	0.05672000
O	1.06730200	1.13842500	0.23936400

Int7

S	-1.02929300	0.16158100	0.07096800
O	-1.77809200	-0.14286900	-1.08767000

O	-1.20592000	-0.59995600	1.24908900
H	0.81684700	-1.86007300	-0.55403200
H	1.87359900	1.27946000	0.01007200
O	-0.30249000	1.37996900	0.13551200
O	2.45818500	0.52090600	-0.15844300
O	1.75183100	-0.51676900	0.39114000
O	0.79876600	-0.89186600	-0.60356900

Int8

S	0.82280900	-0.04282100	-0.00769300
O	1.71964700	0.31534900	1.02217800
O	1.09271800	-1.01994500	-0.98065300
H	-2.95969000	0.27397300	0.67963400
H	0.17686800	1.95750300	-0.17401400
O	0.32971300	1.22183800	-0.78316700
O	-2.36032500	0.43663800	-0.06270000
O	-1.57283200	-0.70090700	-0.07061400
O	-0.50668500	-0.44626600	0.82713900

Int9

S	-0.71328800	-0.17682100	0.01326600
O	-1.72114800	-0.41910900	0.96918600
O	-0.15320200	-1.17790600	-0.81378600
H	1.96245900	-0.97438700	-0.56818500
H	-1.70880900	1.61014800	-0.48079500
O	-1.14948600	0.97534100	-0.95003300
O	0.46514100	0.50926600	0.85954500
O	1.56859500	0.74576000	-0.01537800
O	2.38497100	-0.35918100	0.05505600

Int10

H	-0.87166400	1.51370800	-0.22016300
S	1.29176500	-0.10755400	-0.21924500
O	0.92827200	1.19353100	-0.73754200
O	1.07980600	-0.09143300	1.34926000
H	0.20118400	0.26541400	1.57781300
O	0.04697200	-1.15229700	-0.62119900
O	-1.10174800	-1.04557000	0.19597500
O	-1.90376200	-0.04359200	-0.29067200
O	-1.54925900	1.13207900	0.37296300

Int11

H	-1.82470400	-1.41574400	-0.71878600
S	0.99203000	-0.08779700	-0.43353400

O	1.53781800	1.23334500	-0.49515600
O	1.99313200	-1.03148400	0.37024300
H	2.37840800	-0.55152900	1.12140800
O	-0.07140900	-0.08224000	0.89289800
O	-1.12330500	0.79844400	0.57276800
O	-2.23894100	0.02700600	0.37839400
O	-2.15056800	-0.52356600	-0.90240700

Int12

H	2.43432800	-1.23693200	0.54818300
S	-1.35181300	0.13514300	-0.04674000
O	-1.14802700	-0.80657800	-1.10810400
O	-0.85414200	-0.52529900	1.30661700
H	-0.03655300	-1.02690900	1.12637400
O	-0.13457500	1.31268400	-0.19372300
O	1.10589300	1.00060200	0.37112500
O	1.79093800	0.16926300	-0.49117900
O	1.64381700	-1.13797700	-0.00057600

Int13

H	3.20494100	0.04197200	0.01090700
S	-1.38914600	0.01212000	-0.30120800
O	-1.52772000	-0.69692800	0.93891600
O	-0.93392800	1.50472600	0.02594200
H	-0.26369100	1.50955900	0.72858600
O	0.05546400	-0.53510500	-0.99025600
O	0.92372100	-1.06979200	-0.03623400
O	1.46779000	-0.02195900	0.68563500
O	2.42531000	0.60087600	-0.11402400

Int14

S	0.34379900	-0.10323700	-0.08031400
O	0.92440300	-0.93869700	-1.07870100
O	0.46154300	-0.55016600	1.42200400
H	-0.28065400	-1.13799400	1.62417000
H	2.24490800	0.71697800	-0.43872700
O	1.49341200	1.00097500	0.09414500
O	-1.25066600	-0.82848100	-0.20224500
O	-1.87986400	0.42164400	-0.02563100
O	-0.68195800	1.15382500	-0.19712500

Int15

S	-0.60234800	-0.16300000	-0.46060000
O	0.19556700	1.11975900	0.25145800

O	-0.84694700	-1.20219100	0.71485100
H	-1.63928900	-0.93036200	1.20008200
H	-2.27093300	1.29180000	-0.59894100
O	-2.01883300	0.63567700	0.05864500
O	1.59309900	0.98117500	0.16043600
O	1.78446400	-0.37711900	0.34408700
O	0.98612400	-0.87648000	-0.68341900

Int16

H	-2.67441500	0.42365400	0.93707000
S	-1.09409600	-0.29905400	-0.30001300
O	-2.62046500	-0.08217000	0.11069900
O	-0.74339700	1.28645600	-0.36056700
H	0.19458600	1.38537700	-0.13444500
O	-0.36791900	-0.79937300	0.84719200
O	1.85957500	-0.44648000	-0.98787700
O	2.36133900	-0.28414000	0.11997400
O	2.00903700	0.69768600	0.77027700

TS1-2

S	-0.87522200	-0.01963600	-0.19268700
O	-2.27162600	0.08019800	-0.46605100
O	-0.25850400	1.32662700	0.17565800
H	0.81244400	1.20489000	0.28668200
H	-1.12093300	-1.61387200	1.20833000
O	-0.65052000	-0.76863200	1.20852500
O	1.06503100	-0.62545000	-1.04466100
O	1.82498900	-0.57679600	-0.04887300
O	2.07963600	0.65444700	0.37390000

TS2-P

S	0.68805100	-0.10368000	-0.06847900
O	1.88480600	-0.58965100	-0.63955100
O	-0.08269600	-1.06624600	0.74327400
H	-1.32649400	-0.92119000	0.59902700
H	1.75258100	1.55856200	0.65975100
O	1.03014800	1.00766200	0.98972700
O	-0.21801900	0.52917700	-1.06659900
O	-1.80733200	0.70131400	-0.09751800
O	-2.23627000	-0.45456700	0.05027600

TS2-3

S	-0.79833400	0.05600600	-0.06921400
O	-1.79588400	-0.93366500	0.07022300

O	-1.01626900	1.27995400	-0.72723900
H	1.89719700	0.99736000	0.61711800
H	-0.22438500	-0.37194700	1.91982600
O	-0.19488800	0.40510400	1.34259600
O	0.47560400	-0.66616800	-0.79204900
O	1.62545100	-0.73932600	0.01791100
O	2.29355300	0.46391300	-0.09013400

TS3-4

S	-0.81335000	-0.13364700	-0.01621900
O	-1.00010500	-0.46440500	1.34946400
O	-1.90608200	0.25615400	-0.81996200
H	1.33079400	1.09675300	0.01795800
H	-0.09701700	1.76144700	0.96860500
O	0.13841500	1.35307200	0.12403300
O	0.18887300	-0.94011100	-0.74420500
O	1.80285800	-0.66913700	0.24024200
O	2.24851800	0.37444500	-0.24045300

TS4-P

S	-0.26881700	-0.00079900	0.06361500
O	-0.66250100	1.35536700	0.14568800
O	-1.05865300	-0.94778200	-0.61640300
H	1.48670000	-0.31429900	0.36323600
H	1.59214900	0.94369400	-0.98451400
O	1.33322500	0.03379900	-0.78372800
O	0.54070800	-0.51846100	1.20487300

TS5-6

S	-1.34484600	-0.00101300	-0.23183100
O	-1.82741500	0.00116600	1.11694200
O	-0.51422300	1.21598400	-0.59089900
H	0.52001000	1.27499000	-0.09967900
H	0.52416900	-1.27421100	-0.09724400
O	-0.51277200	-1.21773500	-0.58744000
O	1.65888100	-1.08268500	0.37053400
O	2.09957500	0.00178300	-0.19312600
O	1.65512300	1.08341500	0.37226600

TS6-7

S	1.06646500	-0.13738600	0.03637000
O	1.05855600	-0.86185400	-1.17151700
O	1.71934400	1.11365000	0.12657300
H	-0.86524500	1.70916700	0.44148400

H	-1.57321400	-1.14939300	0.73402100
O	0.55344700	-0.72928100	1.22094800
O	-1.97341700	-0.89158900	-0.11101600
O	-2.20230000	0.45802100	0.03105000
O	-0.98375300	1.11585400	-0.31571500

TS7-8

S	-0.87983700	-0.06824200	-0.09110800
O	-1.56673600	-0.62881400	1.01087900
O	-1.31154900	1.13727100	-0.68493600
H	1.42535800	-0.51700800	0.90302200
H	1.46690700	-0.96417800	-0.68155700
O	-0.14940200	-0.98896600	-0.94148700
O	2.14128600	-0.54048700	-0.04872000
O	1.63806900	0.80359200	-0.07709900
O	0.64647200	0.53903500	0.89589600

TS7-9

S	-0.84997400	0.16924000	0.06212900
O	-1.58229300	0.61941900	-1.05238400
O	-0.24379400	1.08284000	0.95869000
H	1.83556900	1.21936300	0.32923500
H	-0.17504800	-1.52042600	0.03362000
O	-1.25036200	-1.14761000	0.62783300
O	0.56271300	-0.81102400	-0.63039400
O	1.72545400	-0.61530100	0.16856800
O	2.28066400	0.57082700	-0.24192700

TS7-P

S	-0.83556400	0.09830400	0.08120800
O	-1.85522600	0.27256000	-0.88038800
O	-1.06503800	-0.70471700	1.22751300
H	0.14878700	-1.80524300	-0.61099500
H	1.35207800	1.11939800	0.23059100
O	0.01031400	1.27802800	0.29544200
O	2.21236800	0.53611000	-0.11764000
O	1.83857400	-0.61771800	0.17278400
O	0.34252800	-0.87514000	-0.81257700

TS8-4

S	-0.88021400	0.12271800	0.07113400
O	-1.78789300	0.11678300	-1.01014700
O	-1.13714400	-0.61178000	1.25123800
H	2.57106200	0.62755200	-0.82748100

H	1.39781900	1.07750500	0.20593200
O	-0.06568500	1.32070300	0.22751000
O	2.28554800	0.55953700	0.09577800
O	1.46906400	-0.96032900	0.08544600
O	0.50042600	-0.88348100	-0.71440000

TS8-9

S	-0.72851900	-0.02624900	-0.13137900
O	-1.92918700	0.68326000	0.09396000
O	-0.48774700	-0.80593200	-1.27383000
H	1.95764400	-0.94830900	0.75775000
H	-0.87176000	-0.57258300	1.89643400
O	-0.43527300	-0.94844700	1.11917600
O	2.01250900	-0.56822500	-0.13209000
O	1.79021000	0.76748000	0.06823300
O	0.37078900	1.11447500	0.05553600

TS9-P

S	0.68806100	-0.10371100	0.06842200
O	1.88470900	-0.59017700	0.63935500
O	-0.08261300	-1.06586700	-0.74382100
H	-1.32676500	-0.92119700	-0.59921600
H	1.75281000	1.55877500	-0.65895800
O	1.03031100	1.00820600	-0.98909200
O	-2.23642300	-0.45450100	-0.05020400
O	-1.80715200	0.70118400	0.09740600
O	-0.21820900	0.52887800	1.06678400

TSR-14

S	0.53963900	-0.08140300	-0.11478600
O	1.28270500	-0.98140200	-0.93962200
O	0.58059700	-0.48279400	1.42848300
H	-0.09920200	-1.15675800	1.58279300
H	2.35172300	0.99345900	-0.19880900
O	1.43791000	1.21883900	0.03468100
O	-1.50480400	-0.91242800	-0.34183000
O	-1.90591000	0.19391000	0.21847000
O	-1.25134100	1.14709200	-0.34360700

TSR-15

S	-0.66958200	-0.08772800	-0.42729100
O	0.03771000	1.14553000	0.20166500
O	-0.73645500	-1.21254700	0.69302100
H	-1.45510500	-0.99479400	1.30485700

H	-2.48144500	1.15436500	-0.51312600
O	-2.20039300	0.42438200	0.05016800
O	1.72243000	0.93862000	0.24993700
O	1.82474500	-0.32739400	0.32462000
O	1.18319700	-0.81308200	-0.76379500

TS15-P

S	-0.62627600	0.09120500	-0.33786200
O	0.05603100	-0.57338500	0.88593700
O	-1.99868400	-0.66009500	-0.46144500
H	-2.40642100	-0.82770200	0.40455800
H	-0.44761100	2.00087200	0.52872800
O	-1.19439800	1.40166700	0.36655200
O	0.89814600	-1.52153200	0.23256600
O	2.16398400	0.01164500	-0.48464000
O	1.68422900	1.01264500	0.02009400

TS14-P

S	0.39086400	0.11225400	0.05562200
O	0.68008500	1.39310400	0.57760000
O	0.25637400	-0.04488600	-1.48997400
H	1.00922100	-0.55968100	-1.81925500
H	2.37535000	-0.15644200	0.72041400
O	1.78064900	-0.67807000	0.16561500
O	-1.62219700	0.70569600	-0.10938600
O	-1.87081600	-0.55313200	0.09200400
O	-0.42889600	-0.95770600	0.79025100

TS14-2

S	0.45779500	-0.09943300	-0.06024800
O	0.82417000	-0.85690300	-1.19294200
O	0.21399600	-0.75919000	1.29006500
H	-0.78489300	-1.01647500	1.08950300
H	2.38785900	0.64045600	-0.36183000
O	1.71664000	0.75058700	0.32519300
O	-1.57786200	-0.71538800	-0.16375400
O	-1.81510500	0.65475000	-0.03822800
O	-0.47779900	1.17201200	-0.19079700

TS5-10

H	-0.68736700	1.23190100	0.00381400
S	1.28015700	0.07523400	-0.37721700
O	0.46204500	1.34465400	-0.34847100
O	1.63480200	-0.19868300	1.15904300

H	0.82774600	-0.34109800	1.67762000
O	0.37259900	-1.06091400	-0.74782800
O	-1.22845200	-1.08414000	0.30629400
O	-2.00884500	-0.25132900	-0.15486600
O	-1.81001100	0.98859400	0.33008200

TS10-12

H	-2.27066100	1.69367800	-0.17964100
S	1.34385600	-0.16198200	-0.09853400
O	1.18099600	0.94917100	-0.98837000
O	0.91635300	0.28244300	1.36275700
H	0.10622800	0.82284100	1.32516100
O	0.06025500	-1.23969600	-0.39799000
O	-1.13622400	-1.00523100	0.30547400
O	-1.88870700	-0.12667900	-0.39087300
O	-1.54982900	1.14939000	0.16287900

TS12-13

H	2.65875300	0.92718400	-0.33037100
S	-1.32121400	-0.10802300	-0.10765000
O	-1.21045700	-0.04965000	1.32157000
O	-0.84703900	1.28220000	-0.71648700
H	0.00844800	1.57467500	-0.35445100
O	-0.15374500	-1.11534400	-0.71365200
O	1.24017500	-1.08952700	-0.30714200
O	1.49905400	-0.18053300	0.66174100
O	1.78104000	1.05616800	0.05487300

TS10-11

H	-1.98311300	0.83957000	-1.19252600
S	1.09716600	0.36389500	-0.38383800
O	0.78342500	1.17268900	0.75511800
O	2.34574200	-0.55808700	-0.02162400
H	2.30285100	-0.84613700	0.90463100
O	-0.05044400	-0.90004800	-0.41245100
O	-0.96817900	-0.80711800	0.64411000
O	-2.18052500	-0.49486900	0.08101100
O	-2.16431800	0.86046300	-0.24250200

TS13-4

H	2.13052600	1.63529400	0.61651100
S	-1.23846400	-0.00842400	-0.19467600
O	-1.76680200	-0.13328000	1.12978200
O	-0.58170000	1.34355800	-0.45380800

H	0.52524400	1.30914400	-0.32323500
O	-0.42442600	-1.07235500	-0.78638300
O	1.48160200	-1.23346000	-0.13435700
O	1.64872600	-0.32825300	0.68954700
O	1.78755800	1.07258400	-0.09208900

TS11-P

H	-0.92708800	1.05123600	-1.41770200
S	0.74121400	0.25085300	-0.14672900
O	0.95665800	0.65779500	1.20257100
O	2.08349400	-0.61310600	-0.49566100
H	2.63263400	-0.71144200	0.29791400
O	-0.32679400	-1.08862000	-0.22861700
O	-1.44926100	-0.73626200	0.56242400
O	-2.26830500	-0.04058300	-0.10016500
O	-0.69141400	1.27659400	-0.50712100

TS16-P

S	-0.95430700	0.00000000	-0.28104400
O	-2.39429300	-0.00005500	-0.46103700
O	-0.63875700	1.23877400	0.66245700
H	-1.46575500	1.53799600	1.07041900
H	-1.46570600	-1.53812800	1.07032900
O	-0.63868400	-1.23876500	0.66246200
O	1.04349500	0.00006600	-0.86058700
O	1.85619100	0.00008900	0.32839100
O	3.04709600	-0.00009300	-0.03719200

HSO_3^-

S	-0.17756600	0.03959900	-0.37844700
O	-0.04678400	1.37215800	0.25545200
O	1.33951900	-0.62505000	0.06826800
H	1.59376100	-0.06984600	0.81608300
O	-1.13682200	-0.81757600	0.33116400

HSO_4^-

S	0.14184500	0.03027000	-0.00560800
O	0.90207800	-1.19098500	-0.06654100
O	0.15954600	0.83554400	-1.20926100
H	-1.95961600	0.24728100	0.04603000
O	0.30191600	0.79992400	1.21025700
O	-1.40227700	-0.53593400	0.07100700

Int1´

S	-0.80413400	-0.15460000	-0.08149800
O	-1.88113600	-1.08472100	-0.46884000
O	-1.63195500	1.31501400	-0.20333200
H	-2.55485700	1.03908600	-0.26379300
O	-0.43621300	-0.23195900	1.33329800
O	2.02557800	-0.98142600	-0.41773000
O	2.08062600	0.05552500	0.26662500
O	1.77072400	1.10688000	-0.31405100

Int2´

S	0.77900500	0.07450300	-0.05299400
O	1.70339700	0.22459100	1.03343200
O	0.15725000	1.30344100	-0.52259800
H	-1.65337900	1.08701000	-0.27634400
O	1.16452200	-0.82674600	-1.10123800
O	-0.48317200	-0.68810100	0.76213800
O	-1.58656500	-0.74455900	-0.10279800
O	-2.30676900	0.44649200	0.07159400

Int3´

H	-0.20899000	1.28762400	0.14847200
S	1.48117300	-0.00279800	-0.26861500
O	0.70363900	1.45635800	-0.15352100
O	1.76107100	-0.39539600	1.11617500
O	0.43125200	-0.86651200	-0.88139000
O	-1.48714900	-1.06580600	0.36905200
O	-2.22035100	-0.24504900	-0.16866300
O	-2.12468500	0.96104800	0.23701700

Int4´

H	-0.89896700	1.30676700	0.08618600
S	1.31797400	0.03303200	-0.29314500
O	0.61693300	1.33232900	-0.46046900
O	1.66290800	-0.21864600	1.09959400
O	0.02183500	-1.11285100	-0.62830300
O	-1.03086700	-1.07402700	0.30549100
O	-1.99308000	-0.20042800	-0.17470100
O	-1.80130700	1.04421200	0.43390600

Int5´

H	-1.12807200	1.11588300	-0.82019300
S	1.07424500	0.33490700	-0.14953100
O	1.42546900	0.56626700	1.24144600
O	2.07580500	-0.38618600	-0.92243700

O	-0.09711700	-1.06982800	-0.04068700
O	-1.21573500	-0.71165200	0.70883100
O	-2.18851100	-0.29391700	-0.18275500
O	-2.00739300	1.08601600	-0.40281300

Int6′

S	-0.70788100	-0.30267600	-0.32219900
O	0.13625200	1.06866400	0.32866700
O	-0.99719800	-1.21845900	0.75726600
H	-1.98535000	1.47530500	-0.62588800
O	-2.12026100	0.68711600	-0.09114400
O	1.51030200	1.07781700	0.03447500
O	1.94008900	-0.22438700	0.29504500
O	1.19474600	-0.96981300	-0.60167600

TS1-2′

S	-1.20713600	-0.25869500	-0.24704100
O	-2.62779300	-0.08647200	0.08032700
O	-0.61174300	1.32255700	-0.41099400
H	-0.37631900	1.57229400	0.49367700
O	-0.43524800	-0.67950100	0.94989900
O	1.93796800	-0.63976000	-0.97163300
O	1.99015500	-0.38215200	0.22892500
O	2.20797100	0.78618000	0.55584900

TS2-P′

S	-0.74902500	-0.01654700	0.01668000
O	-1.85026300	0.36433300	-0.81323300
O	-0.01232800	1.26901800	0.47710700
H	1.02455000	1.12287700	0.38646900
O	-1.04685300	-0.82539700	1.15854900
O	0.30345500	-0.69711700	-0.88994900
O	1.73264800	-0.70085800	0.01704200
O	2.24332400	0.48275500	-0.03118400

TS1-P′

S	-0.81083900	-0.09652700	0.01221100
O	-1.87118500	-1.05530500	-0.31852200
O	-1.51379900	1.33968800	-0.49593500
H	-2.37376900	1.06228100	-0.83340600
O	-0.55702300	0.10773200	1.42761300
O	1.36925200	-0.93882200	-0.55888600
O	2.07768400	-0.27573400	0.22418600
O	2.41347000	0.88271000	-0.19870300

TS3-4′

H	-0.45420100	1.26480500	-0.03959100
S	1.34070700	0.00410200	-0.27969300
O	0.51667000	1.35831200	-0.39633300
O	1.74034500	-0.16026300	1.11574300
O	0.32164800	-1.05853300	-0.68886300
O	-1.27965100	-1.08149600	0.34410700
O	-2.05568900	-0.23114800	-0.14354200
O	-1.86796100	1.00682300	0.33322300

TS4-5′

H	-1.15002200	1.26375700	-0.50051800
S	1.07036200	0.27389700	-0.29713000
O	0.89335300	1.13402500	0.86886500
O	2.32928200	-0.45421700	-0.34826600
O	0.04429100	-1.17511300	0.13960100
O	-1.18188100	-0.81153900	0.68325000
O	-2.01251000	-0.40190800	-0.35984000
O	-2.06950800	1.00298900	-0.32678500

TS5-2′

H	-0.90690700	1.00527400	-1.30580700
S	0.96415300	0.18982000	-0.08632000
O	1.15119000	0.43222900	1.32022100
O	2.04942300	-0.51965600	-0.73643200
O	-0.38934900	-1.09143600	-0.19834000
O	-1.48093400	-0.71581800	0.53504500
O	-2.30914300	0.00541600	-0.15897500
O	-0.83612900	1.38396500	-0.42565400

TSR-6′

S	-0.88640400	-0.34521800	-0.23893100
O	0.03857200	0.30617900	0.92346500
O	-1.92657500	-0.96691000	0.56346600
H	-1.85236700	1.54417600	-0.01227400
O	-1.52999000	1.05975600	-0.78586900
O	1.22671100	0.95403200	0.27530600
O	2.22479600	0.03283900	0.24758700
O	1.97084000	-0.88848300	-0.74455700

TS6-P′

S	-0.62647500	-0.30003500	-0.24721900
O	0.24522900	1.06997600	0.31551200

O	-0.96117700	-1.11344000	0.89672900
H	-1.97940500	1.43318200	-0.77758700
O	-2.10352000	0.75199000	-0.10871400
O	1.64188200	0.93108800	0.08621700
O	1.97812100	-0.35042900	0.25873300
O	0.69984000	-0.86826300	-0.85684100
